# Rest

**Published:** 1877-12

**Authors:** Edwin T. Darby


### REST.

BY PROF. EDWIN T. DARBY, D.D.S.

The average American, engaged in the active pursuits of life, needs, and his system demands, periods of rest and recreation.

It is impossible for man, constituted as he is, to follow incessantly one train of thought or toil continuously in a given direction without injury to both the physical and mental system—nay, without insanity. The fact that we are an overworked people, both in mind and body, is proverbial, and with each year the truth is becoming more apparent.

That nervous diseases are upon the increase, no one who has given the matter a moment's thought can deny. Consider for a moment the sudden deaths from heart disease, from apoplexia; the wrecks of mind and body which meet us on every hand; the care-worn faces and tottering gait which we see in early manhood, but which belong only to later life. Is this because we are physically and mentally inferior to other nations? No, on the contrary, experience has shown that under favorable circumstance the American people are both physically and mentally equal to any of the old world, and the impaired health of our people is due almost entirely to the rush with which they pursue their business callings, accompanied with high living and insufficient rest and recreation. There seems to be something in our climate which incites men to activity, and activity so intensified that we rush through life, keeping (as it were) the engine of our being at the highest rate of speed for the time, not considering that the machine which runs fastest must of necessity wear out soonest. There are few subjects upon which men engaged in active business life will not talk with more reason than that of rest as the means of promoting health. The excitement of business becomes almost a disease, and it is not until the system begins to succumb to the strain put upon it that men realize or admit that it is possible for them individually to fall victims to this disease of overwork.

What has been said of general business life is eminently true of professional life, and especially so of the profession of Dentistry. There are few men in the profession, who have been in regular practice for ten or more years, who are healthy men. Do you not, by a moment's reflection, recall a score of professional friends who are dyspeptics? whose nervous system in more or less shattered; whose mind is just a little off balance; who, in short, is a little the worse for his ten years' service?

Is Dentistry an unhealthy occupation? Followed as it ought to be, it is not. Followed as it is by the majority of men who are so fortunate (or unfortunate, perhaps,) as to have a full practice, it is.

The dentist who works an hour or two at the chair in the morning, and an hour or two in the laboratory in the afternoon; who takes ample time for his meals, and rests after they have been eaten; who leaves his office at three or four o'clock in the evening and drives in the park or digs in the earth, may plod on for years, perhaps a score of them, and show no signs of nervous disorder, nor experience any symptoms indicative of failing health. To such an one, and followed in such a way, the practice of Dentistry is not unhealthful. But look again; in yonder city is your professional brother who enters his office at eight or nine o'clock in the morning, and stands at his chair incessantly engaged in the most delicate of manipulations, and upon the teeth of the most sensitive organizations, until four or six o'clock in the evening, only stopping to partake of his mid-day meal, which ordinarily is but a light lunch. He does this not one day only, but every day from Monday morning until Saturday night, week after week, and month after month. Followed in this way, Dentistry, *is* an unhealthy occupation.

But, you ask, is it the close confinement that break him down in health? The mechanic, the watch-maker, the tradesman is confined within doors equally as much, and yet shows less signs of failing health. Is it the hours spent in work that makes the dentist so tired when night approaches?

The farmer may commence an hour or two earlier in the morning, and keep it up an hour or two later in the evening, but he is not fatigued to the same extent. He works equally hard, but he works in a different atmosphere; he works in the sunlight, surrounded by the beauties of nature. He works in the open air develops muscle, affords an abundance of oxygen for his lungs, and makes him a healthful, happy man. It is not work that kills men; it is the incessant strain upon our sympathies while inflicting pain upon our voluntary victims. It is the responsibility, which every conscientions man feels, to do the best thing possible for those who seek his services. It is the work of hands, and mind, and heart. It is the effort to keep one's self in one grand equipose—the effort to be courteous, and gentle, and kind, when we feel nervous, and cross, and perplexed. It is the daily routine of care, the constant thinking of the same things day after day, week after week, and year after year.

It has been estimated that one-fortieth of the dental profession become insane, and as many more dissipated; and at times I do not wonder that such is the case. I recall at this moment the history of two men, eminent in their day and generation, who commenced the practice of Dentistry within a few miles of the beautiful lake upon whose borders I sit to-day. The first, after an eventful life, a large and lucrative practice of many years, became dissipated and insane, and died by his own hand. The other, removing from a country village to the metropolis of our nation, enjoyed for years the confidence of many, and secured to himself a large and wealthy practice. Of a highly organized nervous temperament, he felt his health giving way under incessant work, and resorted to stimulants, which with him soon became a necessity. With bad habits once formed, he went from bad to worse, and finally died a miserable drunken pauper in that city's almshouse.

Nor are similar histories rare.

That the practice of Dentistry is peculiarly trying to the nervous system I think none who have given it a few years trial will deny. It is a constant drain upon the nerve force, and requires on the practitioner's part the best care to avoid disastrous consequences. Each day takes from us more than night can give, and by this exhaustive process we soon approach the night of death.

How, then, is the dentist to preserve his health and render himself best fitted to perform his work and prolong his life?

First: By a systematic arrangement of his time, and, when once arranged, not to deviate from it. I have the profoundest admiration for the man who has courage enough to lay aside his instrument and cease work at three or four o'clock in the afternoon.

In my opinion such an one will reap a larger harvest in the end than his neighbor who stands at his chair an hour or two longer each day. There should be periods for rest, and these should be daily. The dentist should spend an hour or two at least of each day in the open air, and, if possible, in the sunlight. He should walk, or ride horseback, row in a boat, dig in the earth, play base ball, or do something which will be as complete a change as possible from his daily work. He should engage in something which will change the current of his thoughts; something that will bring into action different muscles of the body; something, in short, totally and entirely different from his professional life.

Second: The dentist should have an annual vacation of at least a month. Eleven months is long enough to pursue one train of thought, one round of duty. He should, if he be a city practitioner, go to the country, where new scenes will be presented to his eyes, new thoughts occupy his mind, and new food delight his palate. Let him spend a few weeks in the Adirondack wilderness, sleeping on hemlock boughs in her forests of pine and hemlock and spruce; fishing in her limpid streams; chasing her bounding roebucks, and eating her delicious venison and trout. Or let him go to old ocean, and sail on her restless billows, bathe in her ceaseless breakers, and sleep that sleep which is restful.

When once the careworn, nervous, dyspeptic dentist has gone forth amid scenes like these; when he has mingled with nature in her grandest forms, he has put himself in an atmosphere of health; and, whether sleeping or walking, he is drinking in that which rejuvenates his wasted energies, restores the balance of his mental capacities, and refits him for the duties of future years. After a month thus spent, he returns with new desires and new purposes. He enters upon his practice better prepared to perform his operations thoroughly; a better man, physically, mentally and morally, for the rest and recreation he has taken.

